# Lipid accumulation impairs natural killer cell cytotoxicity and tumor control in the postoperative period

**DOI:** 10.1186/s12885-019-6045-y

**Published:** 2019-08-20

**Authors:** Seyedeh Raheleh Niavarani, Christine Lawson, Orneala Bakos, Marie Boudaud, Cory Batenchuk, Samuel Rouleau, Lee-Hwa Tai

**Affiliations:** 1 Department of Anatomy and Cell Biology, Université de Sherbrooke, Pavillon sur la Recherche Appliqué du Cancer at 3201 rue Jean-Mignault, Sherbrooke, QC J1E 4K8 Canada; 2 Department of Pediatrics, Division of Immunology, Université de Sherbrooke, Sherbrooke, QC Canada; 3 Department of Biochemistry, Microbiology and Immunology, University of Ottawa, Ottawa, ON Canada; 4 Centre de Recherche Clinique de Centre Hospitalier de l’Universite de Sherbrooke, Sherbrooke, QC Canada

**Keywords:** Fatty natural killer cells, Perioperative immunosuppression, Immunometabolism

## Abstract

**Background:**

Natural killer (NK) cell dysfunction following cancer surgery has been shown to promote metastases. Recent studies demonstrate an emerging role for lipids in the modulation of NK cell innate responses. However, the mechanisms involved in lipid modulation of NK cell postoperative anti-tumor function are unknown. This current study will determine whether the lipid accumulation via scavenger receptors on NK cells is responsible for the increase in postoperative metastasis.

**Methods:**

Lipid content in mouse and human NK cells was evaluated by flow cytometry. NK cell scavenger receptor (SR) expression was measured by microarray analysis, validated by qRT-PCR and flow cytometry. NK cell ex vivo and in vivo tumor killing was measured by chromium-release and adoptive transfer assays, respectively. The mediating role of surgery-expanded granulocytic myeloid derived suppressor cells (gMDSC) in SR induction on NK cells was evaluated using co-culture assays.

**Results:**

NK cells in surgery-treated mice demonstrated increased lipid accumulation, which occurred via up-regulation of MSR1, CD36 and CD68. NK cells with high lipid content had diminished ability to lyse tumor targets ex vivo. Adoptive transfer of lipid-laden NK cells into NK cell-deficient mice were unable to protect against a lung tumor challenge. Granulocytic MDSC from surgery-treated mice increased SR expression on NK cells. Colorectal cancer surgical patients showed increased NK cell lipid content, higher CD36 expression, decreased granzyme B and perforin production in addition to reduced cytotoxicity in the postoperative period.

**Conclusions:**

Postoperative lipid accumulation promotes the formation of metastases by impairing NK cell function in both preclinical surgical models and human surgical colorectal cancer patient samples. Understanding and targeting the mechanisms underlying lipid accumulation in innate immune NK cells can improve prognosis in cancer surgical patients.

**Electronic supplementary material:**

The online version of this article (10.1186/s12885-019-6045-y) contains supplementary material, which is available to authorized users.

## Background

Surgical resection is the cornerstone of treatment for solid tumors. Despite complete resection with curative intent, many patients die from recurrent or metastatic disease because minimal residual disease and micrometastases are present at the time of surgery [1–4]. Our research [5–7] and others [1, 2, 8–10] have demonstrated that major cancer surgery initiates a series of physiological responses to promote tissue repair and wound healing. However, the proangiogenic, growth factor replete and immunosuppressed state in the surgically stressed host creates a prometastatic environment that can be exploited by micrometastatic tumors [3].

Natural killer (NK) cells play a key role in the innate elimination of malignant cells. The defective function of NK cells in cancer contributes greatly to tumor escape and metastatic disease [5, 11–14]. Impaired NK cell activity following cancer surgery has been shown in human patients [1, 3, 5, 15] and preclinical studies [5, 16, 17]. Postoperative NK cell suppression is associated with increased metastases in mouse models of spontaneous [5, 18] metastases, while in translational human studies, impaired NK cell function is associated with a higher rate of cancer recurrence and mortality [1, 9, 10, 19]. There is a growing body of literature in the emerging field of perioperative immunology that is characterizing the mechanisms of cancer surgery-induced immunosuppression and immunotherapeutic strategies to prevent cancer recurrence [7, 9, 20, 21].

We confirmed the in vivo role of NK cells in mediating tumor removal following cancer surgery in mouse models of melanoma [5], colorectal [22] and breast carcinomas [5, 7]. Using an adoptive transfer strategy of surgically stressed and control donor NK cells into NK cell-deficient recipient mice, we demonstrated that surgically stressed NK cells cannot protect from a subsequent lung tumor challenge in these recipient mice. Additionally, we ensured that perioperative anesthesia and analgesics did not contribute to immunosuppression [21]. In colorectal cancer surgical patients, functional profiling of peripheral blood mononuclear cells (PBMC) revealed that NK cell cytotoxicity was significantly reduced following primary tumor resection [5]. We recently reported that postoperative NK cell dysfunction is linked to granulocytic myeloid derived suppressor cell (gMDSC) accumulation and Arginase I (ARG1) production [7]. MDSCs have been shown in many disease contexts (cancer, burn injury, chronic inflammation, etc.) to impair immune effector cells through immune suppressive molecules such as ARG1 and nitric oxide synthase [20, 23–26]. During our flow cytometric investigations into the effect of surgical stress on NK cell function, we inadvertently observed accumulation of lipids in NK cells isolated from surgery-treated mice. Further investigations revealed that this lipid-laden phenotype was associated with postoperative NK cell dysfunction. These findings prompted us to investigate the potential role of lipid accumulation on NK cell dysfunction in cancer.

Lipids are a diverse group of low molecular weight molecules that contain fatty acid groups. They represent a major structural component of cellular membranes, participate in inter- and intra-cellular signaling, and serve as important energy sources [27]. Previous animal and human studies have shown the negative effect of dietary lipids on innate immune cell function [27, 28]. These data suggest that lipids could be important mediators of immune cell function. For example, excess lipid accumulation in the liver has been shown to negatively impact macrophage function leading to inflammation, tissue damage and chronic liver disease [29]. The ability of lipids to participate in the innate immune inflammatory response is an emerging field of study, mainly focused on the treatment of inflammatory diseases [29, 30]. However, the mechanisms involved in lipid accumulation and modulation of innate immune function are poorly understood.

The detrimental effect of lipid accumulation in conventional Dendritic Cells (cDC) has been reported [31, 32]. The authors demonstrated that cDC in tumor bearing mice and in cancer patients had increased levels of lipid content. Mechanistically, it was reported that upregulation of scavenger receptors was responsible for lipid accumulation in cDC. Importantly, cDCs with high lipid content were not able to effectively stimulate allogeneic T cells or present tumor-associated antigens [31]. In lipid rich environments, NK cells have been recently reported to rapidly accumulate intracellular lipid droplets and undergo metabolic reprograming towards lipid metabolism. Mechanistically, these NK cells displayed suppressed effector function through downregulation of perforin and granzyme mRNA transcripts and significantly reduced IFNγ production. Moreover, lipid bearing NK cells failed to eradicate tumor growth in vivo, suggesting that lipid accumulation induces NK cell metabolic paralysis and impaired antitumor responses [33]. In another recent study focused on characterizing NK cells for adoptive immunotherapy, ex vivo IL15 treated NK cells displayed an exhausted phenotype with reduced IFN-γ production, reduced CD107a degranulation and significant upregulation of fatty acid metabolism [34]. An increase in lipid accumulation and fatty acid oxidation (FAO) metabolism in tumor-infiltrating myeloid-derived suppressor cells (T-MDSCs) were found to enhance inhibitory activity towards T-cell proliferation, supporting tumor growth and microenvironment development [35]. Aside from these reports, there are no studies on NK cell association with lipids and the impact of this interaction on NK cell antitumor activity. While the role of lipid accumulation and metabolism has been recently outlined in NK cells, the metabolic state of NK cells under cancer-surgery induced immunosuppression is unknown. Here, we report the novel finding that following primary tumor resection, postoperative NK cells express up-regulation of three scavenger receptors (SR) and increase lipid accumulation with negative effects on NK cell-mediated tumor recognition and removal.

## Methods

### Mice

C57BL/6 (B6), BALB/c and NK cell-deficient mice (IL2γR-KO) were purchased from The Jackson Laboratory. Female mice aged 6–8 weeks (20-25 g) were used for all experiments, where 4–6 mice were used per experimental group according to previous publications [36]. Animals were housed in pathogen-free conditions at the Central Animal Care Facility of the Université de Sherbrooke (Quebec) and the Animal Care Veterinary Service facility of the University of Ottawa (Ontario) with access to food and water ad libitum. Animal were euthanized by cervical dislocation under anesthesia (isoflurane). All studies performed on animals were conducted in accordance with university guidelines and the Canadian Council on Animal Care. The protocols were approved by Animal Care Committees at both universities.

### Cell lines and viruses

Murine B16F10LacZ melanoma cell line was obtained from Dr. K Graham (London, Ontario) and maintained in complete DMEM (cDMEM). Cells were resuspended in media without serum for intravenous (iv) injection through the lateral tail vein. 3 × 10^5^ cells at > 98% viability were injected in 0.1 ml per mouse. Murine CT26lacZ colorectal carcinoma, murine 4 T1 breast carcinoma, murine YAC-1 lymphoma and human K562 leukemia cell line were purchased from ATCC and maintained in complete RPMI (cRPMI). All cell lines were verified to be mycoplasma free and show appropriate microscopic morphology at time of use.

### Establishment of murine surgical stress model

The murine surgical stress model was conducted as previously described [5, 22]. Routine perioperative care for mice was conducted as per standard protocols including the use of Buprenorphine (0.05 mg/kg) for pain management and isoflurane for induction and maintenance (2.0–2.5%). Surgical stress was induced in mice by an abdominal laparotomy (4 cm long midline incision) and left nephrectomy preceded by an iv challenge of 3 × 10^5^ B16lacZ cells or CT26lacZ to establish pulmonary metastases. Animals were euthanized at 3 days following tumor cell injection and their harvested lungs were stained with X-gal (Bioshop) as described previously [22]. Total number of surface visible metastases was determined on the largest lung lobe (left lobe) using a stereomicroscope (Leica Microsystems) by a research technician blinded to treatment groups.

### Antibodies and flow cytometric analysis

To analyze splenic lymphocyte populations, organs were removed from mice and RBCs lysed using ACK lysis buffer. Fc block was added prior to antibody staining. The following mAbs were used: anti-TCRβ (H57597), anti-CD122 (TM-beta1), anti-NK1.1 (clone), anti-MSR1 (REA148), anti-CD36 (HM36) and anti-CD68 (FA-11) were purchased from eBiosciences. Isotype controls were purchased from BD Biosciences. BODIPY 495/503 was purchased from Life Technologies (D3922). The following human antibodies were used: anti-CD3 (SK7) anti-CD56 (NCAM), CD36 (CB38), granzyme B (GB11) and perforin (δG9), from BD Biosciences. Flow cytometry acquisitions were performed on a CytoFLEX 30 Summit instrument (Beckman Coulter). Data was analyzed with CytExpert software. Bioimaging was performed using a fluorescence microscope (Leica).

### Microarray and qPCR

NK cells were sorted on a FACS Aria (BD) by the Flow Cytometry Core of the University of Ottawa. Cells were double sorted to > 95% purity. RNA was extracted from the sorted and pooled NK cells using an RNeasy Mini Kit (Qiagen #74106) used in conjunction with QIAshredder Columns (Qiagen #79656). The quality of all samples was validated by using a Bio-analyzer 2100 (Agilent Technologies Inc.). 100 ng of purified RNA was loaded onto GeneChip Mouse Gene 1.0 ST arrays according to manufacturer instructions. CEL files were later processed by AltAnalyze V2.0 under default parameters. A detection above background score > 70 and a pV < 0.05 were used to filter probe sets. Gene expression was evaluated using constitutive probe sets shared across splice variants. Gene enrichment analyses were performed on the subset of genes induced (*n* = 223) or repressed (*n* = 109) over 2 fold by surgical stress. All p values reported have been corrected for multiple hypothesis testing using the Benjamini–Hochberg method.

The following primers were used for validation of microarray results by qPCR.

Msr1 Forward: GACAGAGAATCAGAGGCTCT.

Msr1 Reverse: AAGGACTTCAACTTCTCCTG.

CD36 Forward: GATGACGTGGCAAAGAACAG.

CD36 Reverse: TCCTCGGGGTCCTGAGTTAT.

CD68 Forward: AGTCTACCTGGACTACATGG.

CD68 Reverse: TGCATTTCCACAGCAGAAGC.

The following primers were used for validation of NK cell purity results by qPCR.

Eomes Forward: CGGTGTGGAGGACTTGAATGA.

Eomes Reverse: AATCCGTGGGAGATGGAGTT.

Gata3 Forward: TCCTCTACGCTCCTTGCTACT.

Gata3 Reverse: AGGAGGGTTTAGGGAGGAAAGA.

Tbet Forward: CTGGAGCCCACAAGCCATTA.

Tbet Reverse: TTGGAAGCCCCCTTGTTGTT.

RORc Forward: AACCAGTATCCTGTTCCCAGC.

RORc Reverse: TGTCGCCACTGGAAGGATAG.

ID2 Forward: CGGTGAGGTCCGTTAGGAAAA.

ID2 Reverse: TGACGATAGTGGGATGCGAG.

ID3 Forward: TGTGGGGACAAGTCATCTGG.

ID3 Reverse: TGGTAGCTGCCCATCTGAGAG.

### Fatty acid phagocytosis assay

5 × 10^6^ purified NK cells (NK cell isolation kit II, Stemcell) were resuspended in 500 μL of 10 μg/mL fluorescently labeled (Bodipy FL) palmitic fatty acid (Molecular Probes) at 37 °C for 15–30 min. Controls consisted of unloaded NK cells and NK cells loaded at 4 °C. Flow cytometry acquisitions were performed on a CytoFLEX 30 Summit software (Beckman Coulter). Data was analyzed with CytExpert software.

### NK cell adoptive transfer experiments

For NK cell transfer experiments, splenocytes were isolated from no surgery control or 18 post-surgery from B6 mice, enriched for NK cells with the NK cell isolation kit II using a Robosep cell sorter (Stemcell). These enriched NK cells were further sorted for BODIPY^high^ NK cells by flow cytometry sorting (FACS Aria, BD). 3 × 10^6^ BODIPY^high^ NK cells as determined by flow cytometry were injected via the lateral tail vein into NK-deficient mice. For all transfers, 3 × 10^5^ B16lacZ tumor cells were injected iv 1 h post immune cell transfer. 3 days post immune and tumor cell injection, lungs of NK-deficient mice were isolated and quantified with Xgal (as described above).

### NK cell cytotoxicity assays

The chromium release assay was performed as previously described [37]. Briefly, splenocytes were isolated from surgically stressed and control mice at 18 h post-surgery from MSR1-deficient mice. Pooled and sorted NK cells were re-suspended at a concentration of 2.5 × 10^6^ cells/ml and then mixed with chromium labelled target cells (Yac-1), which were re-suspended at a concentration of 3 × 10^4^ cells/ml at different effector to target ratios (E:T) (50:1, 25:1, 12:1, 6:1). The cell mixture was incubated for 4 h prior to analysis of ^51^Cr release in the supernatant using a gamma counter (Perkin Elmer).

### Granulocytic MDSC:NK cell coculture assay

gMDSC were purified using the Mouse MDSC isolation kit (Miltenyi). Purified gMDSC were seeded in triplicates in 96 V-bottom well plates at 1:1 ratio with purified NK cells. Following 24 h of co-culture in the presence of absence of a transwell insert (5.0 μm, Corning), NK cells were harvested and stained with anti-MSR1 (REA148), anti-CD36 (HM36) and anti-CD68 (FA-11) for flow cytometric analysis.

### Human PBMC for flow cytometry and cytotoxicity assay

Human whole blood was collected (CIPO study, 2017–1506 approved by the ethics board of CIUSSS de l’Estrie CHUS) and processed immediately for PBMC using Ficoll-Paque (Stemcell). PBMC were resuspended at a concentration of 5 × 10^6^ in freezing media (RPMI with 12.5% Human Serum Albumin and 10% DMSO). 1 mL aliquots were frozen in cryovials overnight at − 80 °C and transferred to liquid N_2_ for long-term storage. Following batch thawing of viably frozen cells, PBMC were stained with anti-human CD3, CD56, granzyme B, perforin and BODIPY 493/503 flow cytometry analysis. For cytotoxicity assessment, PBMC were rested for 20 h with recombinant IL2 (250 U). ^51^Cr-labelled K562 cells were then added and assessment of target cell killing was determined as above.

### Statistical analysis

Statistical significance other than microarray work was determined by student t test with a cutoff *P* value of 0.05. Data is presented as +/− SEM. Prism v.7 was used for all statistical tests.

## Results

### NK cells accumulate lipids following surgery

**Fig. 1:**
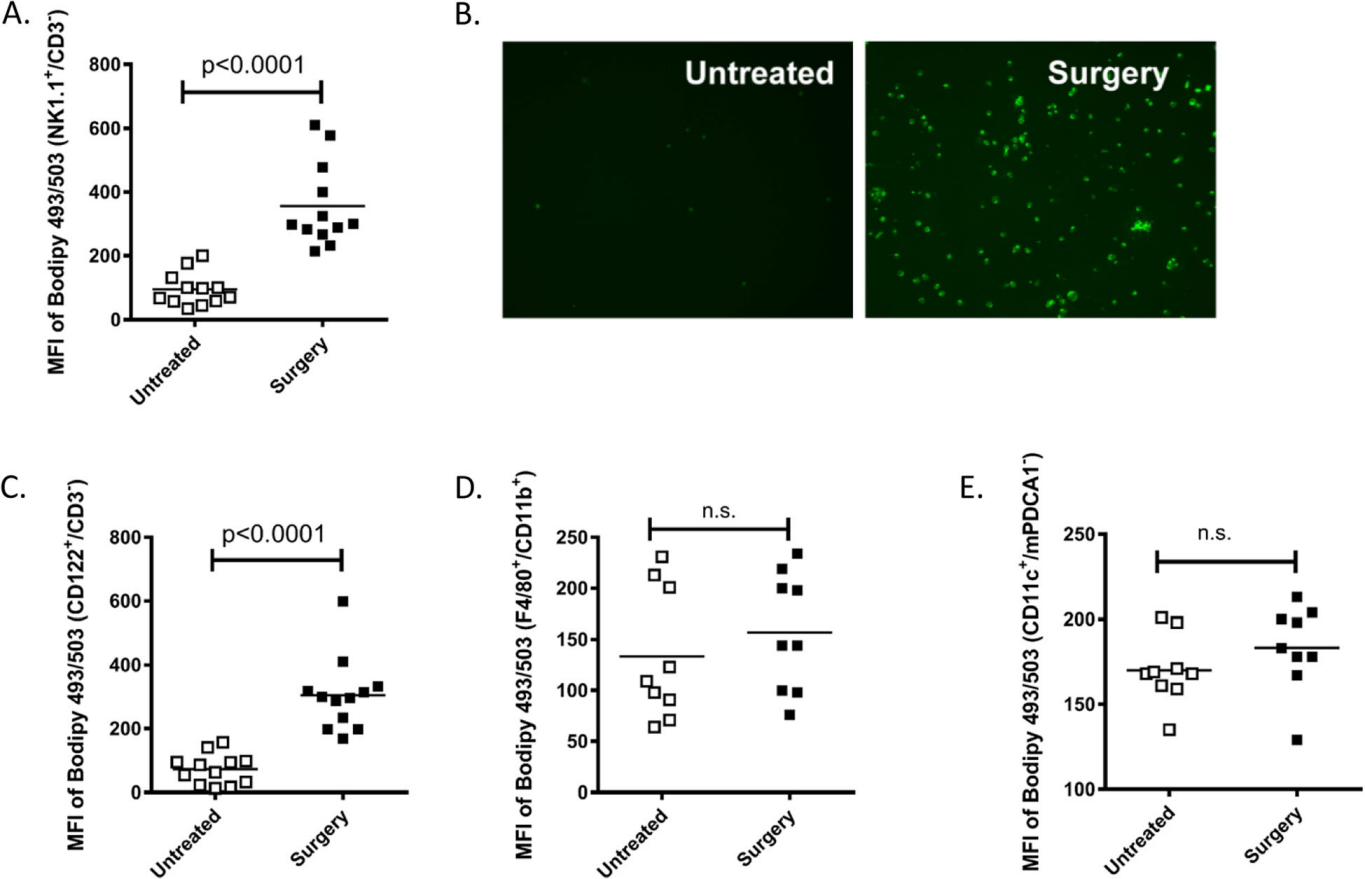
Lipid accumulation in postoperative NK cells. **a** Splenic single cell suspensions from B16 tumor bearing C57Bl/6 (B6-B16) mice were prepared, counted, stained with NK markers (NK1.1^+^, CD3^−^) and BODIPY 495/593 and analyzed by flow cytometry. The Mean Fluorescent Intensity (MFI) of BODIPY^+^ NK cells is shown from surgery-treated and untreated mice. **b** Fluorescent microscopy imaging of purified NK cells from surgery-treated and untreated mice stained with BODIPY 495/503. **c** MFI of BODIPY^+^ NK cells (CD122^+^, CD3^−^) is shown from surgery-treated and untreated mice in CT26 tumor bearing BALB/c mice. MFI of (**d**) BODIPY^+^ macrophages (F4/80^+^, CD11b^+^) and (**e**) conventional dendritic cells (CD11c+, mPDCA1^−^) is shown from surgery-treated and untreated B6-B16 mice. Pooled data are displayed from three similar experiments, *p* values as shown (n.s., no significance)

We previously demonstrated that NK cell antitumor cytotoxic function is critically impaired following cancer surgery and significantly contributes to the growth of lung tumor metastases in the B16 melanoma [5], CT26 colorectal tumor [22] and 4 T1 breast tumor metastasis models [7, 38]. During flow cytometric investigations into the mechanisms of NK cell impairment following surgery, we observed accumulation of lipids in splenic NK cells (NK1.1^+^/CD3^−^) isolated from surgery-treated (abdominal nephrectomy) B16F10lacZ-tumor bearing C57Bl/6 mice (B6-B16) as compared to NK cells from untreated control mice using the lipophilic fluorescent dye Bodipy 493/503 (Fig. 1a). From both flow cytometry and microscopy, we observed increased lipid accumulation in surgery-treated NK cells over controls. We verified these results using fluorescent microscopy to visualize Bodipy^+^ flow cytometry sorted NK cells (NK1.1^+^/CD3^−^) from surgery treated and untreated B6-B16 mice (Fig. 1b). To further support our observations of fatty acid accumulation in NK cells in the B6-B16 model, we assessed fatty acid levels in NK cells from the BALB/c-CT26 model of experimental colorectal cancer and surgery, which we have previously established to study the prometastatic effects of major surgery [5, 18]. In this model, we also observed increased lipid levels in NK cells (CD122^+^/CD3^−^) from surgery-treated mice compared to controls (Fig. 1c). The presence of lipids in innate NK cells prompted us to investigate whether other innate myeloid subsets might display a similar phenotype in the postoperative period. Therefore, we measured lipid content in macrophages and conventional dendritic cells (cDC), comparing surgery-treated and untreated controls in the B6-B16 model. In contrast to postoperative NK cells, no differences in lipid levels as measured by Bodipy 493/503 were observed in macrophages (Fig. 1d) or cDC (Fig. 1e). Taken together, these results suggest surgical stress increases lipid accumulation in NK cells.

### Scavenger receptors are upregulated on NK cells following surgery

**Fig. 2:**
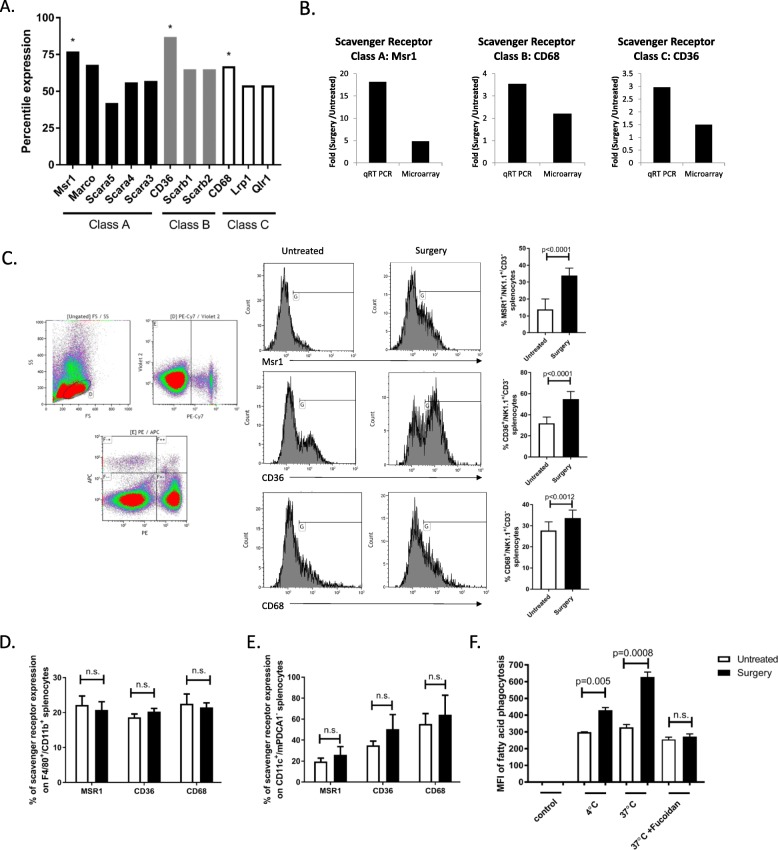
Scavenger receptors are upregulated on NK cells after surgery. **a** Percentile ranking of gene expression for phagocytosis receptors induced by surgery. **b** Fold induction of scavenger receptor expression following surgical stress. Microarray and qPCR were performed on separate runs using identical NK isolation procedure. Both analyses used mRNA pooled from the spleen from 20 untreated or 25 surgery treated mice. Splenic single cell suspensions from untreated and surgery-treated mice were prepared, counted, stained with (**c**) NK markers (NK1.1^+^, CD3^−^) or (**d**) macrophage markers (F4/80^+^, CD11b^+^) or (**e**) cDC markers (CD11c^+^, mPDCA1^−^) and scavenger receptor markers (Msr1, CD36 or CD68) and analyzed by flow cytometry. **f** Phagocytic capacity of purified NK cells from surgery treated and untreated B6-B16 mice cultured with fluorescently labeled palmitic fatty acids. Flow data are representative of at least three similar experiments where *n* = 4–6/group, p values as shown (n.s., no significance)

To investigate the mechanism of lipid accumulation in postoperative NK cells, we performed an unbiased microarray analysis of genes induced by surgery from flow cytometry sorted splenic NK cells (NK1.1^+^/CD3^−^) in the B6-B16 model. To verify the purity of sorted NK cells, we examined the transcription factors Eomes, ID2, RORc, Tbet and Gata3. Our qPCR results show that our surgery treated and control NK cells are ID2^+^/Eomes^+^/Tbet^+^, RORc^+^/Gata3^−^/ID3^−^ suggesting that they are NK cells, and not innate lymphoid cells-1 (ILC1) (that are Eomes^−^), nor ILC2 (that are Gata3^+^), nor ILC3 (that are negative for NK1.1 by cell surface expression and ID3^+^) (Additional file 1: Figure S1). From this microarray data set, we observed an increase in genetic programs related to the metabolic process and phagocytosis in surgery-treated NK cells vs untreated control NK cells. Specifically, we observed an increase in gene expression of phagocytosis receptors from scavenger receptors (SR) classes A, B and C in NK cells following surgery (Fig. 2a). From this data set, the macrophage scavenger receptor Msr1 (CD204), CD36 (Srb) and CD68 displayed the highest percentile ranking. Because SR play an important role in the intracellular transport of lipids, we validated these microarray results by measuring transcript levels of Msr1, CD36 and CD68 on flow sorted NK cells (NK1.1^+^/CD3^−^) from surgery-treated and untreated mice by qPCR (Fig. 2b) and cell surface SR expression levels on whole splenocytes by flow cytometry (Fig. 2c). We observed a significant increase in the expression of each SR on NK cells following surgery compared to untreated controls. Additionally, we measured SR cell surface expression on macrophages and cDC. In contrast to postoperative NK cells, SR cell surface expression level differences were not detected in macrophages (Fig. 2d) or cDC (Fig. 2e) in the postoperative period. To test the mediating role of SR in NK cell lipid accumulation, we isolated splenic NK cells from surgery-treated and untreated B6-B16 mice and cultured them with fluorescence-labeled palmitic fatty acid. We detected a significant increase in palmitic fatty acid levels in surgery-treated NK cells compared to untreated controls (Fig. 2f). Taken together, these findings support that SR play mediating roles in the enhanced uptake of lipids following surgery.

### Lipid-laden NK cells show diminished expression of critical NK cell receptors

**Fig. 3:**
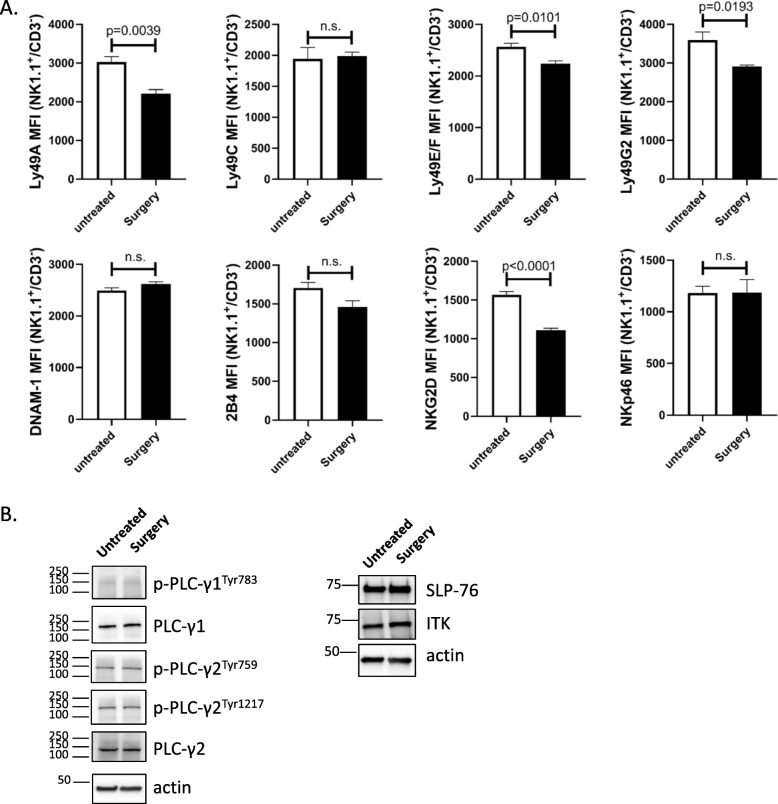
Lipid-laden NK cells show diminished expression of critical NK cell receptors. **a** Splenic single cell suspensions from untreated and surgery-treated mice were prepared, counted, stained with antibodies against various activating and inhibitory NK cell markers and analyzed by flow cytometry. Flow data are representative of three similar experiments where *n* = 4/group, *p* values as shown. **b** Immunoblot analysis measuring the expression of ITK, SLP-76, and PLCγ. β-actin was used as a loading control. Data is representative of 3 independent experiments

Multiple groups have established that NK cells are regulated by the integration of signals derived from activating and MHC-specific inhibitory receptors on their surface. The responsiveness of each NK cell is quantitatively adjusted to ensure self-tolerance while at the same time guaranteeing useful reactivity against potential threats, such as transformed tumor cells [13, 39, 40]. Therefore, we examined the effect of postoperative lipid accumulation on MHC-specific receptors Ly49A, Ly49C, Ly49E/F, and Ly49G2; the signaling lymphocytic activation molecule (SLAM) family molecule 2B4 (CD244), the DNAX accessory molecule (DNAM-1, CD226), the mouse natural cytotoxicity receptors NKp46 and the critical activating receptor NKG2D (Fig. 3a). Comparing NK cells from untreated and surgery-treated B6-B16 mice, we detected a downregulation in NK cell surface expression of Ly49A, Ly49E/F, Ly49G2 and NKG2D, but not in the other receptors. Next, we examined the effect of lipid on NK cell signaling. Specifically, we examined SLP76 (a critical adapter molecule downstream of ITAM-containing surface receptors), ITK (IL-2-inducible T cell kinase), and PLCγ (phospholipase Cγ) (Fig. 3b). Comparing across these 3 important signalling molecules, we did not detect any differences in protein expression in untreated vs. surgery-treated NK cells. These results suggest key NK cell inhibitory (Ly49A, Ly49E/F and Ly49G2) receptors and a critical activating (NKG2D) receptor are affected by surgery.

### Lipid-laden NK cells are impaired in their ability to lyse tumors

**Fig. 4:**
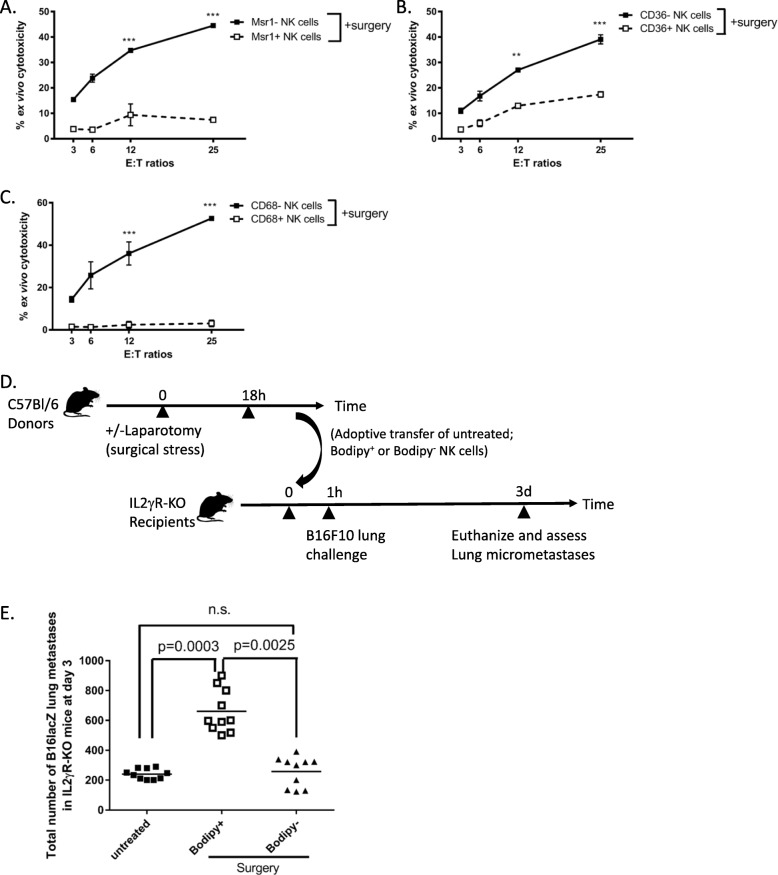
Lipid-laden NK cells are impaired in their ability to lyse tumors. **a**-**c** The ability of purified SR^+^/BODIPY^+^ vs. SR^−^/BODIPY^−^ NK cells, both sorted subsets from surgery-treated mice to kill Yac-1 target cells was tested by ^51^Cr- release assay. The data are displayed as the mean percent (+/− SD) of chromium release from triplicate wells for the indicated effector:target (E:T) ratios. (***, *p* < 0.0001 comparing SR^+^/BODIPY^+^ to SR^−^/BODIPY^−^ surgery-treated NK cells). Data are representative of three similar experiments. **d** Depicts adoptive transfer experimental timeline. **e** Quantification of lung tumor burden at 3d from NK-deficient (IL2γR-KO) mice receiving adoptively transferred 5 × 10^6^ purified NK cells from surgically stressed and control mice, followed with 3 × 10^5^ B16lacZ tumor 1 h post immune cell transfer. Pooled data are displayed from three similar experiments. Treatment groups differed significantly as shown, p values are shown (n.s., no significance)

To investigate whether lipid accumulation in NK cells have functional consequences, Msr1^+^/Bodipy^+^ vs. Msr1^−^/Bodipy^−^, CD36^+^/Bodipy^+^ vs. CD36^−^/Bodipy^−^, and CD68^+^/Bodipy^+^ vs. CD68^−^/Bodipy^−^ NK cells (NK1.1^+^/CD3^−^) were flow sorted from spleens of surgery-treated B6-B16 mice and used as effector cells to lyse chromium-labelled YAC-1 tumor targets in an ex vivo NK cell cytotoxicity assay. We observed that all 3 sets of SR^+^/Bodipy^+^ NK cells from surgery treated mice were responsible for NK cell cytotoxic dysfunction following surgery, while SR^−^/Bodipy^−^ NK cells retained normal cytotoxic activity postoperatively (Figs. 4a-c). These results suggest that SR expression, in general, negatively affects postoperative NK cell cytotoxic function ex vivo. To establish the important role of lipid accumulation in defective NK cell function in cancer in vivo, we adoptively transferred flow sorted Bodipy^+^ or Bodipy^−^ NK cells from donor surgery-treated B6 mice into recipient NK cell-deficient mice (IL2γR-KO). One hour following adoptive transfer of NK cells, we challenged these recipients with intravenous injection of B16F10lacZ lung tumors (Fig. 4d timeline). We have used this model previously to establish the mediating role of NK cells in the increase of cancer metastases following surgical stress [4]. At 3 days post treatment, we found significantly increased lung tumor burden in NK-deficient mice that received Bodipy^+^ surgery-treated NK cells compared to those that received Bodipy^−^ NK cells (Fig. 4e). By transferring Bodipy^+^ surgery-treated NK cells and recreating the effect of surgery on the formation of metastases, our results suggest that the prometastatic effect of surgery is mediated by lipid-laden NK cells.

### Surgery-expanded gMDSC induces scavenger receptor expression on NK cells

**Fig. 5:**
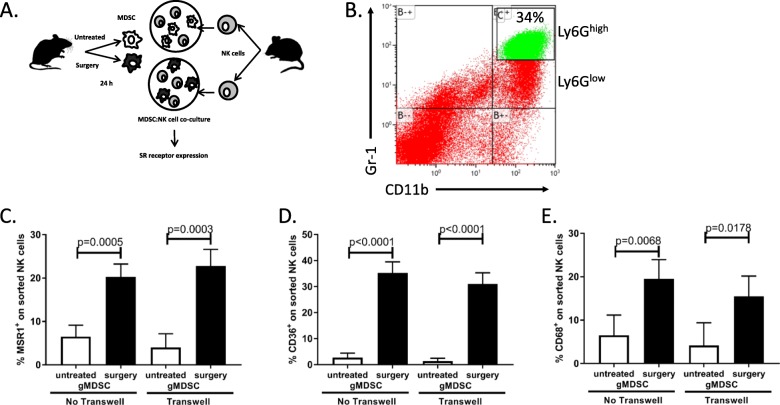
Surgery-expanded gMDSC induces scavenger receptor expression on NK cells. **a** Depicts the experimental set up. **b** Depicts the flow cytometry profile of MDSC used in co-culture assay. **c**-**e** NK cell surface expression of MSR1, CD36 and CD68 by flow cytometry following 20 h of co-culture with MDSC isolated from mice with indicated treatment groups in the presence or absence of transwell inserts. Pooled data are displayed from a minimum of three similar experiments. Treatment groups differed significantly as shown, p values are shown (n.s., no significance)

In our previous studies, we have assessed for surgery-induced tissue signals that might be responsible for the suppression of NK cells. Specifically, we observed an increase in serum IL5, IL6 and TGFβ in surgery-treated mice compared to controls [5]. We, therefore, questioned whether these cytokines could induce SR expression on NK cells. Following treatment of purified NK cells with recombinant IL5, IL6 and TGFβ, we did not detect any differences in SR expression levels (Msr1, CD36, CD68) in the presence of these cytokines (Additional file 2: Figure S2). Next, we questioned whether surgery-expanded myeloid derived suppressor cells (MDSC), which we have recently documented to impair NK cells [7] could induce SR expression on NK cells. We purified granulocytic MDSC (Ly6G^high^ gMDSC) from surgery-treated and control mice and co-cultured them with naïve purified NK cells (Fig. 5a, b). In the presence of surgery-treated gMDSC, we observed significant increases in all 3 SR expression on NK cells (Fig. 5c-e). Moreover, we repeated the gMDSC:NK cell co-culture experiment in the presence of a transwell insert to evaluate whether the observed upregulation of SR on NK cells by gMDSC was dependent on direct cell contact. In the presence of the transwell, we observed that gMDSC from surgery-treated mice still induced the upregulation of SR on NK cells, suggesting that cell-to-cell contact is not required for this effect (Fig. 5c-e). These results further support our previously published studies that surgery-expanded myeloid regulatory cells impair NK cell function. Importantly, they demonstrate that gMDSC impair NK cells through upregulation of SR expression.

### Lipid-laden NK cells from colorectal cancer surgical patients have higher CD36, but lower Granzyme B expression

**Fig. 6:**
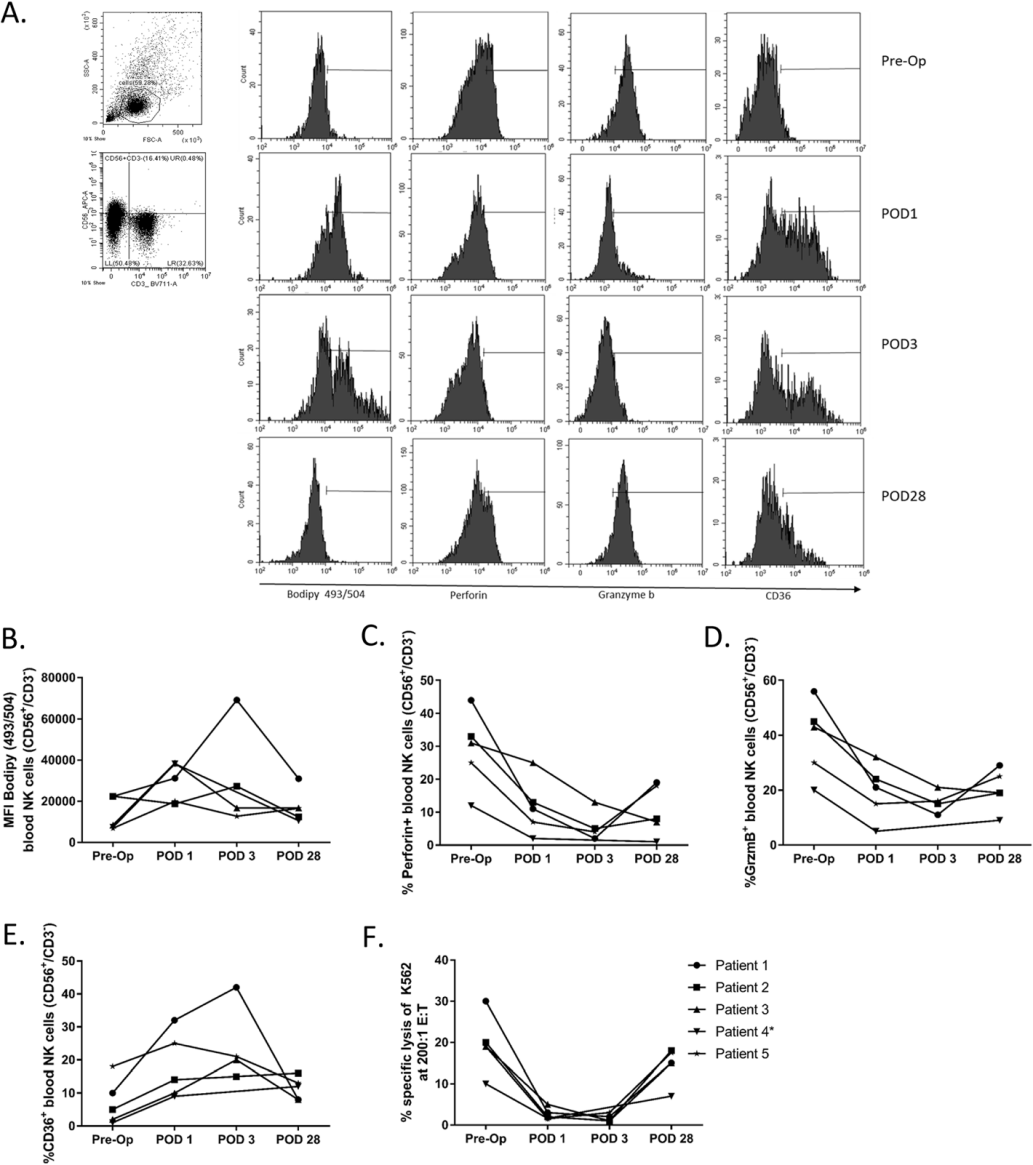
Lipid-laden human NK cells in colorectal cancer surgical patients have higher CD36, but lower Granzyme B and perforin expression and cytotoxicity. **a** Flow cytometry profiles of one representative patient (patient 1 from CIPO study). **b** MFI of BODIPY^+^ human NK cells (CD56^+^, CD3^−^) was evaluated in PBMC from colorectal cancer surgical patients at baseline (preoperative), POD1, POD3(±1) and POD28(±14). Proportion of blood Perforin^+^ (**c**), Granzyme B^+^ (**d**) and CD36^+^ (**e**) NK cells (CD56^+^, CD3^−^) was evaluated in PBMC from colorectal cancer surgical patients at baseline (preoperative), POD1, POD3(±1) and POD28(±14). **f** The ability of PBMC from colorectal cancer surgical patients at baseline (preoperative), POD1, POD3(±1) and POD28(±14) to kill K562 target cells was tested by ^51^Cr- release assay at 200:1 effector cell (PBMC) to target cell (K562) ratio. * Patient 4 is missing POD3

To investigate whether human cancer surgery has the same effect as mouse cancer surgery on NK cells, we measured bodipy levels in NK cells from 5 surgical patients with colorectal cancer. We collected blood from patients enrolled in the “Characterization of Immunosuppression in the Postoperative Period - CIPO” study (Centre Hospitalier Universitaire Sherbrooke, ethics approved study: #2017–1506). Blood was collected at different time points including preoperatively (pre-op), on postoperative day (POD) 1, POD 3 (±1) and POD 28 (±14) (Fig. 6a – 1 representative patient, patient 1 from CIPO study). Following batch thawing of viably frozen down PBMC, we assessed NK cell (CD3^−^/CD56^+^) lipid levels using the lipophilic stain Bodipy 493/503. We observed a consistent increase in NK cell lipid levels at POD1 and POD3 compared to preoperative samples and a consistent reduction to baseline at POD28 in 4 out of 5 patients studied to date (Fig. 6b). Consistent with our findings in mice, we additionally observed higher CD36 (Fig. 6c), lower granzyme B (Fig. 6d), lower perforin expression (Fig. 6e) and reduced cytotoxicity (Fig. 6f) of NK cells from POD1 and POD3 than those from NK cells from the preoperative time point or on POD28. Collectively, our data show that human postoperative lipid accumulation is associated with reduced NK cell cytotoxic effector function.

## Discussion

**Fig. 7:**
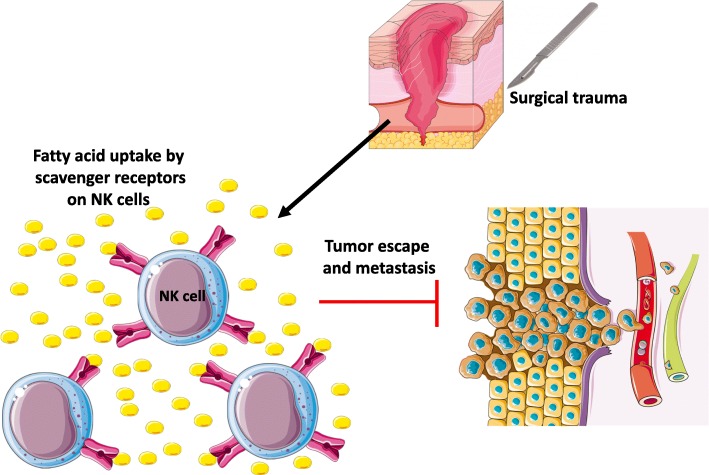
Postoperative lipid-laden NK cells are dysfunctional against tumor targets. Surgical trauma induces up-regulation of scavenger receptors (SR) MSR1, CD36 and CD68 on postoperative NK cells. NK cells with greater SR expression are associated with increased lipid uptake and accumulation. This leads to impaired NK cell anti-tumor immune response and metastatic spread following cancer surgery

This study has demonstrated that a substantial proportion of NK cells in surgically stressed hosts contain increased amount of lipids and this lipid-laden phenotype is associated with enhanced NK cell surface expression of SR and anti-tumor NK cell dysfunction (Fig. 7). These results were obtained using two different preclinical models of surgery and solid tumors (experimental melanoma and colorectal cancer) (Fig. 1), in addition to data collected from colorectal cancer surgical patients (Fig. 6). In particular, these findings were supported by adoptive transfer results showing that fatty NK cells from surgery-treated mice failed to lyse tumors in vivo (Fig. 4).

In this study, we have tried to address two main questions: what is the mechanism of lipid accumulation in NK cells and whether this lipid accumulation has any functional consequences for NK cells. Accumulation of lipids could be due to increased synthesis of fatty acids or increased lipid uptake from plasma. Our data showing increased SR expression on NK cells suggests that the second explanation is more likely (Fig. 2). Scavenger receptors represent a major route in acquiring fatty acids by innate immune cells. Belonging to class A SRs, Msr1 is mostly observed on phagocytic and antigen presenting immune cells, such as DCs (bone marrow and splenic derived) or macrophages, as well as on lung endothelial cells, smooth muscle cells, and liver sinusoidal endothelial [41]. Msr1 recognizes a wide array of self and non-self molecules and has been implicated in the development and progression of various diseases [42]. Additionally, Msr1 plays a major role in the development of cardiovascular diseases and atherosclerosis by increasing cellular lipoprotein internalization. Importantly, the presence of the Msr1 receptor has been linked to the inhibition of DC function through impairment of antigen processing and presentation to T cells, thus negatively regulating the antitumor immune response [31, 43]. Identified as a Class B SR [34], CD36 is present on a large repertoire of cells ranging in function, including DCs, macrophages, microvascular cells, adipocytes, endothelium cells, and skeletal muscle cells [43]. Similar to Msr1, the expression of CD36 on macrophages has been linked to increased uptake of lipoproteins in atherosclerosis [44]. In both animal and human studies, increased CD36 expression in metabolically active tissue has been implicated in regulation of fatty acid metabolism and enhancement of fatty acid internalization and lipid accumulation, thus leading to insulin resistance [45, 46]. Furthermore, in the context of platelet and endothelial cell expression, the receptor has been reported to participate as an adhesion molecule [46]. CD68 is a Class D SR is highly expressed by cells in the monocyte lineage (e.g., circulating macrophages, tissue macrophages and osteoclasts). Functionally, CD68 binds to tissue- and organ-specific lectins or selectins, allowing macrophages to home in on particular targets [43, 47].

The expression of Msr1, CD36 and CD68 has not been reported on NK cells in the postoperative period. Our results indicate that NK cells from surgery-treated mice had preferential up-regulation of SRs, including Msr1, CD36 and CD68. In addition, we demonstrate that accumulation of lipids and up-regulation of SR was observed in surgery-treated NK cells, but not in control NK cells. Furthermore, accumulation of lipids was not observed in cDC or macrophages following surgery, suggesting a unique role of NK cells in this regard. It is likely that fatty acids are transferred to NK cells in the form of modified lipoproteins. Different lipoprotein modifications have been described in cancer and those lipoproteins are likely abundant in tumor-bearing hosts [46]. It is also likely that the accumulation of lipids by NK cells in vitro and in vivo is due to the presence of tumor and host-derived factors that are induced by cancer surgery, which facilitate up-regulation of SR on NK cells. In previous studies, we have shown that surgery increases serum levels of IL5, IL6 and TGFβ [5]. However, ex vivo treatment of NK cells with these cytokines did not induce SR expression (Additional file 2: Figure S2). We also tested whether surgery-expanded gMDSC could induce NK cell SR expression. NK cells co-cultured with gMDSC from surgery-treated mice in the presence or absence of a transwell insert showed higher levels of SR expression compared to untreated controls. Collectively, these results suggest soluble factors likely induce SR expression on NK cells. The molecular mechanism of how MDSC regulates NK cell SR expression and lipid accumulation remain unclear and is currently under investigation in our lab.

In our functional ex vivo and in vivo studies, lipid-laden NK cells had a profound defect in their ability to lyse target tumor cells. One potential explanation could be that lipid-laden NK cells represent immature cells, which are defective in their cytotoxic capacity. We have previously shown that NK cells from surgery-treated mice expressed diminished levels of KLRG1 and CD11b compared to control mice, which suggests they may have lower levels of maturity [5]. The results of the experiment with adoptive transfer of lipid-laden NK cells into NK cell-deficient mice demonstrated the negative consequence of impaired NK cell function on lung tumor clearance. The Lynch group recently reported that NK cells in lipid-rich environments, such as in obese individuals, had impaired trafficking of their cytotoxic machinery to the NK cell-tumor synapse. They showed that this blunted cytotoxicity was due to peroxisome proliferator-activated receptor (PPAR)-driven lipid accumulation in NK cells causing paralysis of both cellular trafficking and metabolism [33]. Whether NK cell: tumor cell synapse formation and cellular metabolism is affected in postoperative NK cells is currently under investigation in our mouse models of surgical stress.

In human studies, we clearly show that an analogous mechanism of surgery-induced fatty acid accumulation is occurring in colorectal cancer surgical patients following their resection. Our human data demonstrate that NK cells accumulate fatty acids at POD1–3 and return to baseline levels at POD 28(+/− 14). Furthermore, these lipid-laden NK cells have higher levels of CD36 and lower levels of granzyme B and perforin expression along with reduced cytotoxicity (Fig. 6). Taken together, these human results demonstrate the importance of metabolic regulation of the immune system in the critical postoperative period. This is likely to impact the ability of innate immune cells to respond to foreign and damaged cells, and may partly explain the increased risk of infection and tumor progression in obese cancer patients undergoing surgical resection.

## Conclusion

In summary, we characterized the biological and clinical significance of lipid accumulation in NK cells in the postoperative tumor microenvironment. Importantly, we demonstrated that NK cell lipid levels increase in colorectal cancer surgical patients following removal of their primary tumor with negative immune functional consequences. Future mechanistic studies on immunometabolic pathways and lipid modulation in NK cells will be important for targeting these critical innate immune lymphocytes to improve surgical cancer outcomes.

## Additional files

Additional file 1: **Figure S1.** Verification of the purity of sorted NK cells. The purity of sorted NK cells was tested by qPCR using the transcription factors Eomes, ID2, RORc, Tbet and Gata3 for qPCR analyses. (PPTX 505 kb)

Additional file 2: **Figure S2.** SR expression on NK cells following treatment with IL5, IL6 and TGFβ. NK cells were treated ex vivo with the recombinant cytokines IL5, IL6 or TGFβ followed by assessment of SR expression on NK cells by flow cytometry. (PPTX 277 kb)

## Data Availability

The datasets used and/or analyzed during the current study are available from the corresponding author on reasonable request.

## Abbreviations

NK: Cells natural killer cells
SR: Scavenger receptors
gMDSC: granulocytic myeloid derived suppressor cells
PBMC: Peripheral blood mononuclear cells
ARG1: Arginase 1
cDC: conventional dendritic cells
FAO: Fatty acid oxidation
t-MDSC: tumor infiltrating myeloid derived suppressor cells
B6: C57Bl/6 mice
ILC: Innate lymphoid cells
ITK IL-2: Inducible T cell kinase
PLCγ: Phospholipase Cγ
CIPO: Characterization of postoperative immunosuppression study
POD: Postoperative day
PPAR: Peroxisome proliferator-activated receptor
