# Supplementary figures and images for: Lipid accumulation impairs natural killer cell cytotoxicity and tumor control in the postoperative period
Source: BMC Cancer. 2019 Aug 20;19:823. doi: 10.1186/s12885-019-6045-y (PMC6701111; doi:10.1186/s12885-019-6045-y)

## Slide 1
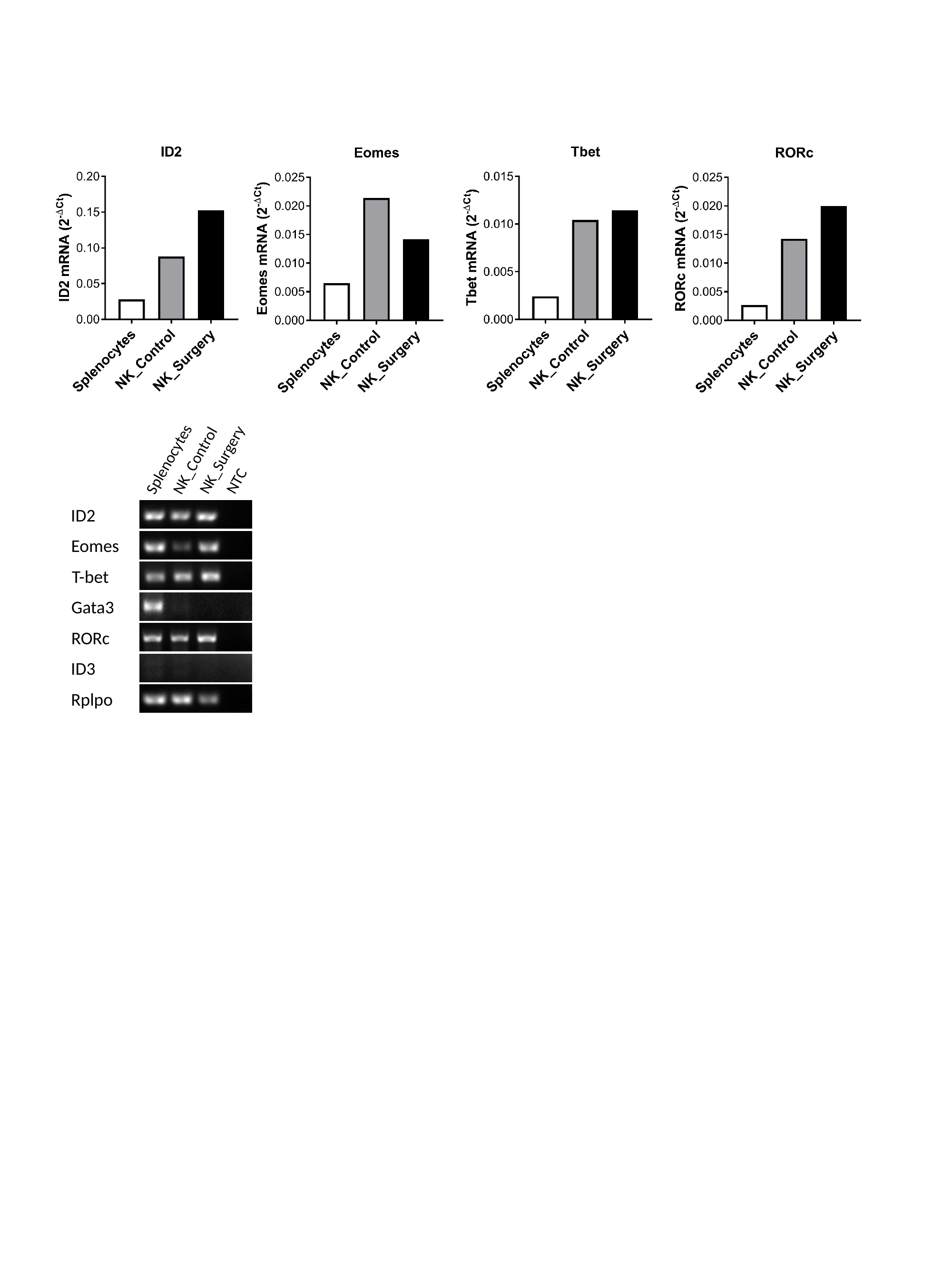

NTC
NK_Surgery
NK_Control
Splenocytes
ID2
Eomes
T-bet
Gata3
RORc
ID3
Rplpo

Supplement: Supplementary file 1 — Figure S1. Verification of the purity of sorted NK cells. The purity of sorted NK cells was tested by qPCR using the transcription factors Eomes, ID2, RORc, Tbet and Gata3 for qPCR analyses. (PPTX 505 kb) [file 12885_2019_6045_MOESM1_ESM.pptx]

## Slide 1
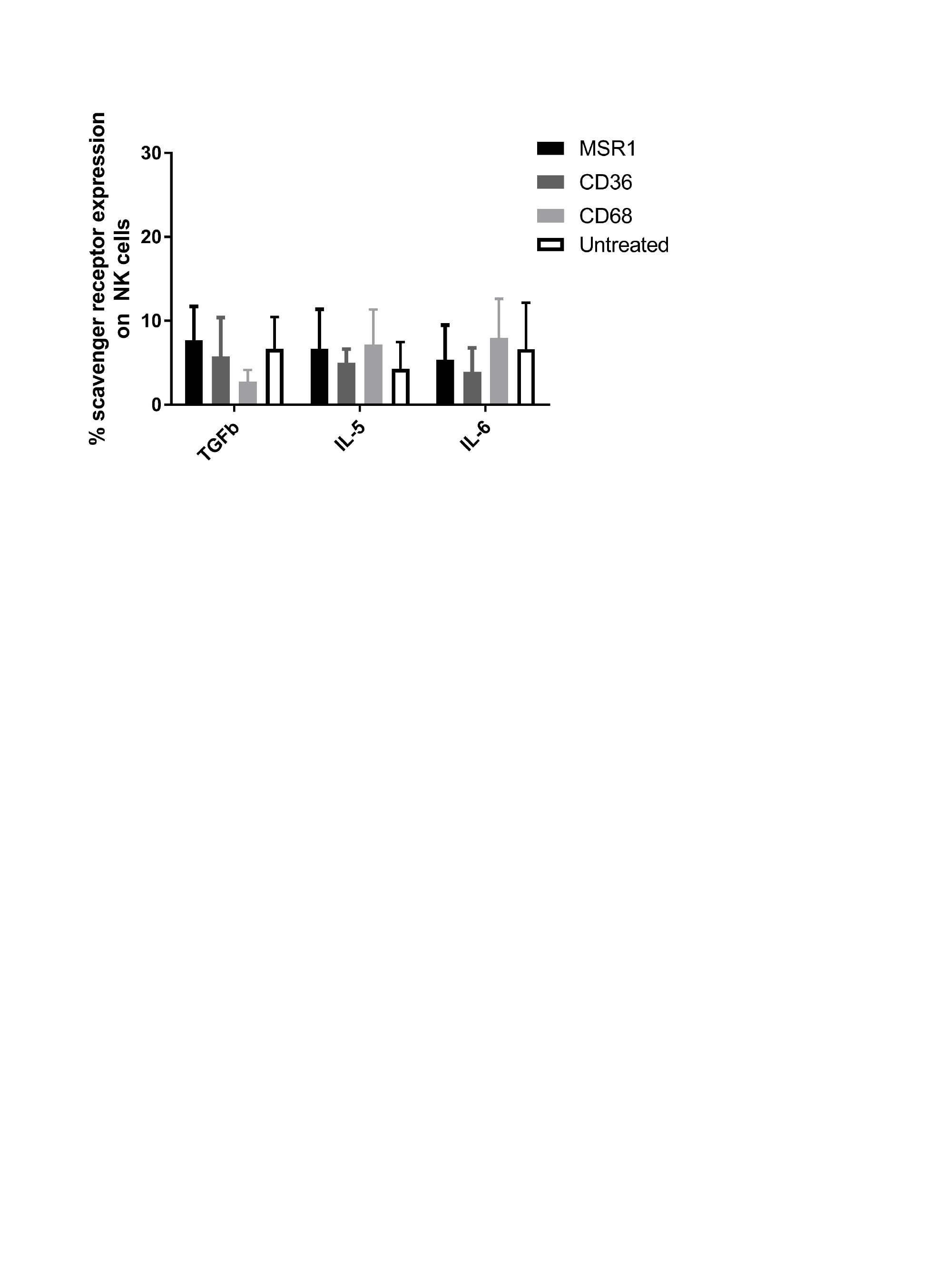

Supplement: Supplementary file 2 — Figure S2. SR expression on NK cells following treatment with IL5, IL6 and TGFβ. NK cells were treated ex vivo with the recombinant cytokines IL5, IL6 or TGFβ followed by assessment of SR expression on NK cells by flow cytometry. (PPTX 277 kb) [file 12885_2019_6045_MOESM2_ESM.pptx]
